# The Durability of Public Goods Changes the Dynamics and Nature of Social Dilemmas

**DOI:** 10.1371/journal.pone.0000593

**Published:** 2007-07-04

**Authors:** Sam P. Brown, François Taddei

**Affiliations:** 1 Section of Integrative Biology, University of Texas at Austin, Austin, Texas, United States of America; 2 University Paris 5, Institut National de la Sante et de la Recherche Medicale, U571, Paris, France; University of Bristol, United Kingdom

## Abstract

An implicit assumption underpins basic models of the evolution of cooperation, mutualism and altruism: The benefits (or pay-offs) of cooperation and defection are defined by the current frequency or distribution of cooperators. In social dilemmas involving durable public goods (group resources that can persist in the environment–ubiquitous from microbes to humans) this assumption is violated. Here, we examine the consequences of relaxing this assumption, allowing pay-offs to depend on both current and past numbers of cooperators. We explicitly trace the dynamic of a public good created by cooperators, and define pay-offs in terms of the current public good. By raising the importance of cooperative history in determining the current fate of cooperators, durable public goods cause novel dynamics (e.g., transient increases in cooperation in Prisoner's Dilemmas, oscillations in Snowdrift Games, or shifts in invasion thresholds in Stag-hunt Games), while changes in durability can transform one game into another, by moving invasion thresholds for cooperation or conditions for coexistence with defectors. This enlarged view challenges our understanding of social cheats. For instance, groups of cooperators can do worse than groups of defectors, if they inherit fewer public goods, while a rise in defectors no longer entails a loss of social benefits, at least not in the present moment (as highlighted by concerns over environmental lags). Wherever durable public goods have yet to reach a steady state (for instance due to external perturbations), the history of cooperation will define the ongoing dynamics of cooperators.

## Introduction

### Social dilemmas

Cooperative or altruistic behaviours have long puzzled biologists [1]–[14]: Given the presence of defectors or cheats, how can more cooperative behaviours persist? The simplest and most common models of cooperation present two interacting players with a simple and symmetric choice, to cooperate or to defect [4]. If they both cooperate, they each receive a reward, *R*, which is larger than the punishment, *P*, obtained if they both defect. If one defects while the other cooperates, the defector receives the ‘temptation’ payoff, *T*, and the cooperator receives the sucker's payoff *S* (methods). This terminology was introduced for the Prisoner's Dilemma, which is defined by the ranking *T>R>P>S*. Given the relative magnitude of the payoff values, a rational player should always defect in one-off encounters, regardless of whether the other player cooperates or not. Thus the problematic outcome is total defection, despite a higher pay-off occurring when everyone cooperates. (maintenance of cooperation in the Prisoner's Dilemma requires additional mechanisms that ensure cooperators are more likely to encounter other cooperators than expected by chance [5]).

The Prisoner's Dilemma represents the strictest form of a social dilemma, however other payoff rankings in the 2-player game are consistent with a social dilemma [10], [15]. The Snowdrift Game (related to the Chicken or Hawk-Dove Game [7], [10], [16]) is defined by the payoff ranking *T>R>S>P*. Thus if the opponent cooperates, it is best to defect (*T>R*). Yet if the opponent defects, it is best to cooperate (*S>P*). The premium on following a distinct strategy is illustrative of negative frequency-dependent selection, ensuring coexistence between cooperators and defectors in a well-mixed population. The Stag-hunt Game (an example of a coordination game [10]) is defined by the payoff ranking *R>T>P>S*. The Stag-hunt Game places a premium on coordinated responses, thus if your partner cooperates, it is best to cooperate (*R>T*), yet if your partner defects, it is best to defect (*P>S*). The premium on coordinated responses is illustrative of positive frequency-dependent selection, ensuring bistability in a well-mixed population (a final outcome of either cooperators-only or defectors-only, dependent on initial frequency of cooperators).

### Public goods

In addition to the study of two-player games akin to the Prisoner's Dilemma, ecological and economic social dilemmas are often couched in the language of public goods (or related notions such as common pool resources or the tragedy of the commons). In contrast to the cooperator games presented above, public goods dilemmas focus attention on an openly accessible ‘good’ or resource that is potentially impacted by the actions of individuals [17]–[20]. Human examples include air quality, scientific discovery or national defence [17]–[20]; microbial examples include siderophores, colicins, signal molecules, extracellular enzymes and polymers [21]–[27]. Public goods raise significant social dilemmas as in particular the lack of excludability from the benefits of the public good ensure that defectors that exploit the public good but make no efforts to generate or conserve it, can prosper.

Whereas the discussion of public goods commonly invokes material resources such as those listed above, theoretical and experimental approaches to the study of public goods effectively replace the public good with a focus on the underlying behaviour of cooperators and defectors [10], [11], [28]. By focusing on the frequency of cooperators, this approach makes the implicit assumption that the public good is entirely defined by the current frequency of cooperators, essentially allowing the ‘public good’ itself to drop out of the analysis, in favour of a study of cooperation versus defection analogous to the two-player models presented above. The ‘cooperation equals public good’ paradigm has been strikingly successful in generating a diversity of approaches to modeling social dilemmas ([1]–[14]; kin selection, group selection, reciprocal altruism, etc).

Here we examine what are the consequences for relaxing the linkage between cooperators and public goods. Details of our modelling approach are presented in the methods section, however the spirit of approach is simple–whereas classic game-theoretic models of cooperation focus solely on the frequency of cooperators *p* (defining public goods implicitly as *p*), we add a second variable *e*, explicitly tracking the amount of public goods. The addition of an explicit public goods variable allows for diverse novel outcomes, for instance transient increases in cooperation in Prisoner's Dilemmas, shifts in invasion thresholds in Stag-hunt Games, and multi-generational oscillations between cooperators and their enduring public goods in Snowdrift Games.

## Results

**Figure 1:**
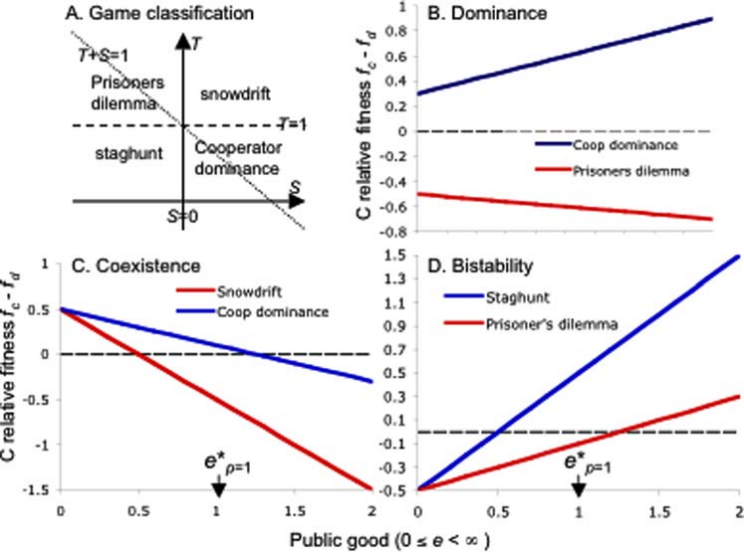
Social dilemmas and payoffs to cooperators and defectors. a, Common social dilemmas organized on the ‘sucker’, ‘temptation’ (*S, T*) plane (*R* and *P* normalised to 1 and 0; see text for details). b–d, expected relative payoff of cooperators as a function of public goods *e* is *f_c_-f_d_* = *S*+*e*(1-*T-S*), for 0≤*e*<∞. Cooperator and defector payoffs are equal on the dashed lines (*f_c_* = *f_d_*). b, Dominance games. Red line, Prisoner's Dilemma, *S* = −0.5, *T* = 1.6. Blue line, cooperator dominance, *S* = 0.3, *T* = 0.4. c, Coexistence games. Red line, Snowdrift Game, *S* = 0.5, *T* = 1.5. Blue line, cooperator dominance, *S* = 0.5, *T* = 0.9 (but becomes snowdrift if *u/c* decreases sufficiently to allow e*_p__ = 1_>*S/*(*S+T*-1), here if *u/c*<4/5). d, Bistability games. Blue line, Stag-hunt Game, *S* = −0.5, *T* = 0.5. Red line, Prisoner's Dilemma, *S* = −0.5, *T* = 1.1 (but becomes Stag-hunt if *u/c* decreases sufficiently to allow e*_p__ = 1_>*S/*(*S+T*-1), here if *u/c*<4/5). Unless otherwise stated, game identities are consistent with *u* = *c.* See methods for more details.

If we normalise the payoffs for mutual cooperation *R* and mutual defection *P* to 1 and 0 respectively, social dilemmas can be described in game theoretic terms by two key parameters, the ‘temptation’ to cheat, *T*, and the ‘sucker’ reward for unilateral cooperation, *S* [10], [29] (Fig. 1a, methods). Traditional game-theoretic analyses focus on the frequency of cooperators, *p* through time, as a function of *S* and *T*. We extend this traditional one-dimensional treatment [10], [11], [29] (methods) with an explicit public-goods equation, tracking the dynamics of the public good *e* (0≤*e*<∞), created by cooperators at a production rate *c*, and lost at a decay rate *u* (incorporating both intrinsic rates of decay and extrinsic rates of removal or dilution),
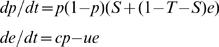
(1)


The pay-offs to cooperators and defectors are now defined by the current intensity of the public good *e* (methods, Fig. 1), which is in turn produced by cooperators. The explicit representation of public good as distinct from cooperators introduces an element of memory into the system, large *c* and *u* (relative to the magnitude of payoffs, *T* and *S*) implies a fast rate of production and decay of the public good, ensuring *p* and *e* are in close agreement; in this case the history of past cooperation is of little importance. In contrast, when *c* and *u* are small, the public good changes only slowly in response to time and current frequency of cooperators, and reflects more strongly the past contributions to the public good. Initially we focus on the special case where *c = u = x*, and look at the affects of varying the general lag parameter, *x*.

### Coexistence: Snowdrift Game

**Figure 2:**
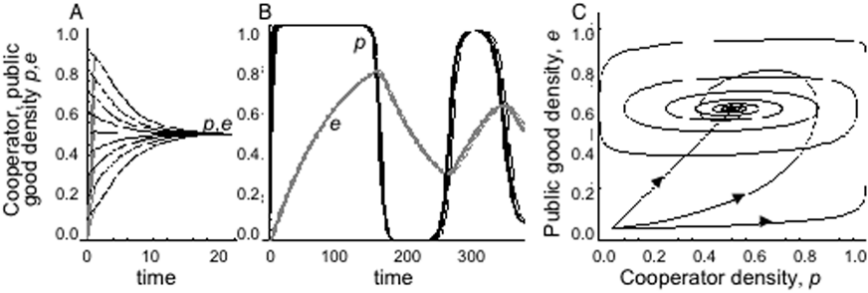
Impact of durability on Snowdrift (coexistence) game dynamics. Snowdrift game (*T* = 1.5, *S* = 0.5, *c = u = x*), stable coexistence of cooperators and defectors at p* = e* = *S*/(*S*+*T*-1) = 0.5, threshold to oscillations *x*<1 (see methods). a, b Temporal dynamics of cooperators *p* (black) and public good *e* (grey). Initial values of *p* range from 0.1 to 0.9. Initial value of *e* is zero. a, *x* = 10. b, *x* = 0.01. c public good (*e*)–cooperator (*p*) phase plane. Lines illustrate simulated trajectories for differing values of *x* (10, 0.1, 0.01) from initial position *p*_0_ = *e*_0_ = 0.05.

In agreement with the traditional one-dimensional model (methods), when *c = u = x* the equilibrium analysis with snowdrift parameters (*T*>1, *S*>0, Fig 1a,c) predicts a stable coexistence of cooperators and defectors at p* = e* = *S*/(*S+T-*1). However, in contrast to the traditional model, the approach towards the coexistence point p* will follow damped oscillations if *x* is sufficiently small (*x*<4*S*(*T*-1)/(*S+T*-1), methods, Fig 2). Decreasing *x* beyond the threshold value acts to strengthen and lengthen the oscillations, allowing both cooperators and defectors to effectively fixate for long periods of time. In Fig. 2b we see that given an initial absence of public good, and despite a predicted 50/50 coexistence point, for the first 250 arbitary time units (potentially many generations), either cooperators or defectors are in near complete domination, irrespective of the starting frequency of cooperators. In Fig. 2c, public good is mapped against cooperator frequency in a phase-plane plot, for differing levels of *x* (summarising Figs 2a,b).

### Bistability: Stag-hunt Game

**Figure 3:**
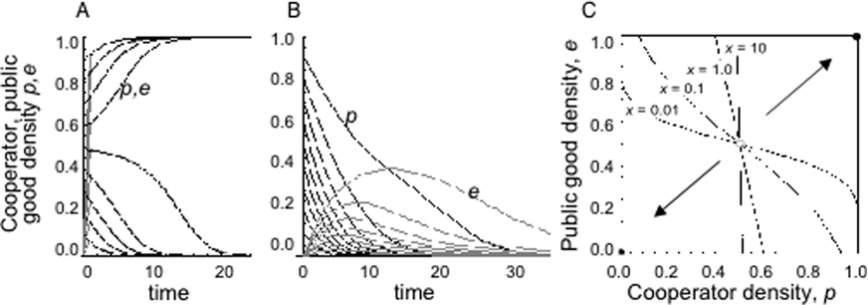
Impact of durability on Stag-hunt (bistable) game dynamics. Staghunt game (*T* = 0.5, *S* = −0.5, *c = u = x*), repellor at p* = e* = *S*/(*S*+*T*-1) = 0.5. a,b Temporal dynamics of cooperator *p* (black) and public good *e* (grey). Initial values of *p* range from 0.1 to 0.9. Initial value of *e* is zero. a, x = 10. b, x = 0.1. c public good (*e*)–cooperator (*p*) phase plane. Lines illustrate simulated separatrices demarcating the basins of attraction for pure cooperator and pure defector equilibria (closed circles). Unstable equilibrium (open circle) at p* = e* = *S*/(*S*+*T*-1) = 0.5. Lines represent differing values of *x* (10, 1, 0.1, 0.01).

Turning to the Stag-hunt Game (*T*<1, *S*<0, Fig. 1a,d), equilibrium analysis predicts bistability (pure cooperation or pure defection at equilibrium) with the watershed or separatrix when *c = u = x* passing through the unstable equilibrium at p* = e* = *S*/(*S+T*-1). In Fig. 3 we illustrate the temporal dynamics of *p* and *e* given that the public good is initially absent (*e*_0_ = 0), for both high and low values of *x*

When equilibration is fast (*x* is large relative to the magnitude of *S* and *T*, fig 3a), we see behaviour close to that predicted from the traditional one-dimensional model, irrespective of the initial public good status, *e*_0_. However, as *x* decreases, and the public good becomes more durable (and slower to produce), we find that the initial condition *e*_0_ has an increasing role in determining the threshold to producer dominance in a Stag-hunt Game. Thus in Fig 3b, we see that despite a predicted threshold at p* = 0.5, we find that even when cooperators are initially numerically dominant (*p*_0_ = 0.9), defectors go to fixation when *e*_0_ = 0.

In Fig. 3c, the dependence on initial conditions of both *p* and *e* is illustrated for varying levels of *x* in a phase-plane plot. When *x* is large, *p* and *e* are intimately tied together, so *p* is a good predictor of *e* and hence of whether cooperators or defectors go to fixation, in keeping with the implicit assumption of the traditional model. In contrast, as *x* drops, *p* and *e* can become dissociated (with *e* being increasingly weighted in favour of past cooperation), so that the current frequency of cooperators *p* is no longer a good predictor of the current strength of the public good *e*, hence it becomes essential to explicitly measure *e* in order to predict the fitness and fate of cooperators and defectors. Thus for example in Fig. 3c we see that when *x* = 0.01, defectors will go to fixation regardless of their current frequency if *e*<0.2, and similarly cooperators will go to fixation regardless of their current frequency if *e*>0.8. Generally, when the lag effect is strong (*x* is small), the history of cooperation becomes central. In the context of invasion biology, the Stag-hunt analysis illustrates the importance of considering whether invaders bring their public goods with them (e.g. microbial supernatent or plant soil).

### Prisoner's dilemma

**Figure 4:**
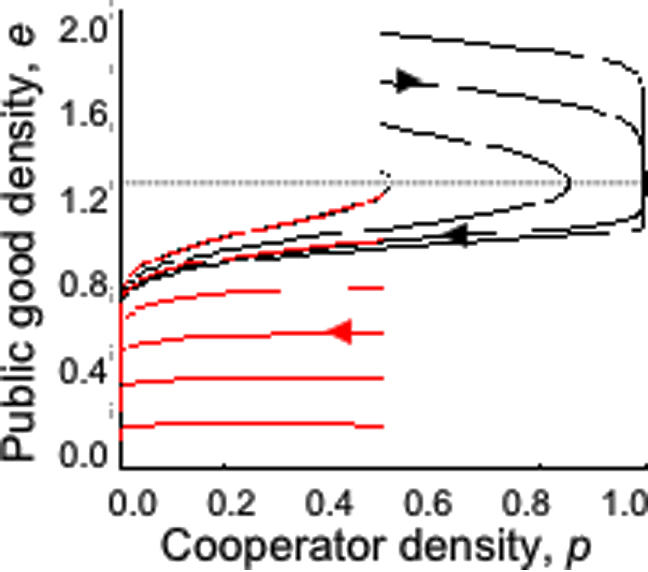
Prisoner's dilemma (defector dominance) game dynamics given durable public goods. Public good (*e*)–cooperator (*p*) phase plane. *T* = 1.1, *S* = −0.5, *c* = *u* = *x* = 0.1. Sole stable equilibrium, p* = e* = 0. Lines illustrate simulated trajectories for differing initial values of *e* (0.2 to 1.2 in red; 1.3 to 1.9 in black) for initial *p* = 0.5

Turning to the Prisoner's Dilemma (*T*>1, *S*<0 Figs 1a,b,d), we find the same conclusion as for the traditional model, ie cooperators are doomed to extinction (sole stable equilibrium is p* = 0, e* = 0). However, when selection against cooperators is weaker for increasing values of the public good (*T*+*S*<1, red line, Fig 1d), then cooperators may transiently increase and even transiently exclude defectors if *e* is sufficiently large (black lines, Fig. 4). Nonetheless, pure cooperation remains unstable as the total production of cooperators when *p* = 1 is insufficient to maintain the public good in sufficient excess. Eventually (with time dependent on *x*) *e* will decline below *S/*(*S+T*-1), and then cooperators will inevitably be sent to extinction in the absence of further external perturbations (Fig. 4).

### Changing the game

The nature of a game (whether a Snowdrift Game or a Stag-hunt Game, etc) will be sensitive to the ecology underlying the game parameters (in our framework, *T, S, c* and *u*). We can now ask what is the affect of a perturbation in the durability parameter *u*, relative to a reference game (pre-perturbation), defined by e*_(*p* = 1)_ = *c/u* = 1 (methods). Changes to the decay of public goods *u* relative to their production *c* (i.e. movement away from *u* = *c*) can change the equilibrium nature of games, by moving the maximal equilibrium value of public goods away from 1 (e*_p = __1_ = 1 when *u = c*, Fig. 1). For instance, increasing the decay rate *u* in a Stag-hunt Game (blue line Fig 1d) reduces e*_p = __1_ below one, to the limit (*u = c*(*S+T*-1)/*S*) where defection becomes dominating and a Prisoner's Dilemma is recovered. Reversing this logic, certain Prisoner's Dilemmas (red line, Fig. 1d) can become Stag-hunts with locally-stable cooperation simply due to an increased durability of the public good (when *u*<*c*(*S+T*-1)/*S*) and therefore a higher stable equilibrium value of public good e*_p = __1_>1, irrespective of the productivity *c* of cooperators. Just as changing the production-decay ratio *c/u* can cause shifts between bistability and defector domination, changing this ratio can also cause shifts between coexistence (Snowdrift Games) and cooperator domination (Fig. 1c). Increasing the decay rate of public good *u* (depressing e*_p = __1_ below one) in a Snowdrift Game (red line, Fig 1c) can lead to the exclusion of defectors, as defectors have insufficient equilibrium public goods e*_p = __1_ to exploit, and so cannot invade. In addition to changing the equilibrium behaviour of games, altering the *c/u* ratio can also modify game dynamics (methods, supplementary materials).

Finally, we note that ‘public bads’ such as pollution, government debt, etc are of course social dilemmas and can be viewed explicitly as the negative space of *e* in our existing models. If we now return to Fig 1b (which has no game changes in positive space of *e*), and extrapolate into the negative space of *e* we see that the blue line (cooperator dominance) has a ‘hidden staghunt’ for sufficiently low *e* (i.e. if the environment is really bad (*e*<*S/*(*S*+*T*-1)), then best to keep on defecting to oblivion), whereas in contrast we find a ‘hidden snowdrift’ for the Prisoner's Dilemma game in Fig 1b (i.e. if the environment is really bad, then best to start cooperating until *e* returns to moderate negative at *e* = *S*/(*S*+*T*-1).

## Discussion

Building on the simplest models of social dilemmas, we have moved from a world consisting only of cooperators and defectors, to a world of cooperators, defectors and their environmental consequences. We present models where the payoffs depend on the state of a public good, that in turn depends on both current and past levels of cooperation. Such an approach is likely to be particularly important in any situation where cooperators and public good are out of steady state equilibrium with each other, as for instance when either genetic or technological innovations or environmental or political perturbations (storms, war) at least transiently displace any equilibrium leading to continued change in both cooperators and public goods. For some examples of cooperation, for instance anti-predator vigilance, the act of cooperating is at least at first sight inseparable from the ensuing rewards. However, there are many more examples of cooperative behaviour where there is a clear separation between the cooperative act and the subsequent generation of reward, with physical intermediates ranging from extracellular microbial enzymes to ant nests to bridges to scientific papers [17]–[27]. By highlighting the capacity for social organisms and their environments to engage in feedback loops, our findings tie social dilemmas into niche construction fields [30]–[32]. We show here that increasing the lag (reducing *x*) between changes in cooperator and public good densities can lead to diverse novel outcomes, for instance multi-generation oscillations between cooperators and their enduring public goods in Snowdrift Games (Fig 2), shifts in invasion thresholds and resulting equilibria in Stag-hunt games (Fig 3) and transient increases in cooperation in Prisoner's Dilemmas (Fig 4).

Replicator dynamics formalism [29], [33] is a convenient and well-studied baseline for a generic treatment of social dilemmas. However, our generic treatment leaves many more dimensions to be addressed–for instance the effects of more complex public goods dynamics, population structure, demography, strength of selection, stochasticity, finite populations, and so on. The dynamics of durable public goods are potentially more diverse than our current model allows, for instance characterized by different patterns of production and degradation as a function of their interactions with cooperators (producers or protectors of a public good) and defectors (non-producers or exploiters of a public good). In more complex scenarios, the public goods (or ‘public bads’ such as pollution or public debt) themselves are subject to independent dynamics due to extrinsic environmental forces (periodic and/or stochastic perturbations), or even to self-replication, making an intriguing bridge between durable public goods and symbionts–both ‘good’ (e.g. crops, livestock) and ‘bad’ (e.g. parasites) (see also [34]). A number of intriguing consequences of playing durable public goods games in a spatially-explicit setting are plausible. Whereas classic approaches to spatially-extended cooperative games have focused on the role of aggregation in cooperators [7], [9], a spatially-extended durable public goods game would focus equally on the role of aggregation in public goods, and would track the degree of concordance between aggregates of cooperators and of public goods to ask in general what conditions would favour aggregations (‘cities’) of public goods? And how resistant are these ‘cities’ against invasion by defectors?

In the absence of any of these potential complications, our generic two state variable model of social dilemmas highlights that dissociating the dynamics of cooperators and public goods has suprising consequences, for instance groups of cooperators can do worse than groups of defectors, if the defectors inherit superior public goods. This notion in turn changes the meaning of defectors or social cheats. In this enlarged view, it is no longer the case that a rise in defectors entails a loss of social benefits–at least not in the present moment (as highlighted by many environmentalist concerns). At best, given sufficiently long-lived public goods, a defector may be a social ‘cheat’ with regards to future generations, having no immediate impact on its social group. Thus an early cheat in a dominant cooperative phase in Fig. 2b has little immediate impact on the rising public good, yet their more numerous strategic descendents can lead to its fall. The recognition that durable public goods are ubiquitous and violate an implicit assumption underpinning all basic models of the evolution of cooperation implies that our work will have diverse consequences across the fields of evolutionary biology, molecular biology, ecology, economics, and political science, and be of practical relevance across many levels of biology, from biotechnology and medicine (concerned with microbial public goods) to the maintenance of environmental services.

## Materials and Methods

Payoffs for cooperation *C* and defection *D* in a Prisoner's Dilemma (and related games) can be expressed in matrix form as follows
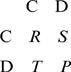
Payoffs are illustrated for the row player, dependent on the strategy of their partner (column player) and themselves. A necessary condition for a social dilemma is that the reward for mutual cooperation is greater than the reward for mutual defection (i.e. *R>P*). Assuming *R*>*P* and normalising to *R* = 1 and *P* = 0, we can reduce the parameter set to *T* and *S* [10], [29], ie the payoff matrix becomes
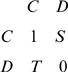



Given this matrix, the expected payoffs for cooperators and defectors as a function of the proportion of cooperators *p* can be simply derived under the assumption of random mixing, ie the probability of interacting with a cooperator is *p* and the probability of interacting with a defector is 1-*p*, regardless of a focal individual's strategy*.* Under the assumption of random mixing, the expected payoffs for cooperators and defectors are *f_c_* = *p+*(1-*p*)*S* and *f_d_* = *pT* respectively, for 0≤*p*≤1.

By relating expected payoffs with fitness, the dynamics of cooperators and defectors in a large well-mixed population can now be described by the replicator equation *dp/dt* = *p*(*f_c_–f*), where *f* is the mean payoff, *f* = *p f_c_*+(1-*p*)*f_d_* (for further details on replicator dynamics and derivation of the replicator equation, see [29], [33]). The replicator equation for 2-player games can now be simplified to
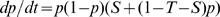



The system has three potential equilibria; pure cooperators (p* = 1), pure defectors (p* = 0) and mixed cooperators and defectors (p* = *S*/(*S+T*-1)). When *T*>1 and *S*<0 (Prisoner's dilemma) only pure defection is stable. When *T>*1 and *S>*0 (Snowdrift Game), only the mixed equilibrium is stable, the system tends towards coexistence at p* = *S*/(*S+T*-1). In contrast, when *T<*1 and *S<*0 (Stag-hunt Game), only the pure equilibria are stable, ie the system is bistable, with p* = *S*/(*S+T*-1) serving as a repellor, separating the basins of attraction for pure cooperation and pure defection.

### Durable public goods

In the above classic framework, the expected payoffs for cooperators and defectors, namely *f_c_* and *f_d_*, are simple and direct functions of the current frequency of cooperators, *p*. We now wish to challenge this framework by assuming that payoffs are directly determined by the extent of a shared public good, *e*, which in turn depends on frequency of cooperators both past and present. We begin by normalizing *e* so that when cooperators (producers of a public good) are fixed in a population for a sufficient period of time to allow the public good *e* to go to equilibrium, this equilibrium is set to one (e*_p__ = 1_ = 1). Likewise, when defectors go to fixation, the public good at equilibrium is set to zero (e*_p = __0_ = 0). Considering that *T* describes the initial payoff of a lone defector in a population of pure cooperators, and conversely, that *S* describes the initial payoff of a lone cooperator in a population of pure defectors, we can define the following payoffs as a function of *e*

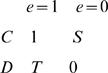



If we again assume that expected payoffs are linear functions of the rewarding element (now *e* instead of *p*), we can now extrapolate between the *e* = 1 and *e* = 0 payoffs, and express expected payoffs as a direct function of the public good, i.e. *f_c_* = *e*+(1-*e*)*S* and *f_d_* = *eT*, for 0≤*e*<∞. These payoff functions are clearly analogous to the classic functions *f_c_* = *p*+(1-*p*)*S* and *f_d_* = *pT*, and are identical under the classic assumption that the public good is determined by the current frequency of cooperators, ie *e* = *p.* Given these expected payoffs, the dynamics of cooperators and defectors in a large well-mixed population (random encounters) can again be described by the replicator equation [10], [33], which now becomes *dp/dt* = *p* (*f_c_–f*) = *p*(1-*p*) (*S*+(1-*T-S*)*e*). To this point, we have specified how public goods *e* influence the dynamics of cooperators, but not the reverse. In order to complete the feedback loop between cooperators *p* and public goods *e*, we consider that cooperators produce public goods at a rate *c*, while public goods decay or are lost at rate *u*, specifying *de/dt* = *cp–ue*, and returning us to model (1). Note that our specification that when cooperators dominate (*p* = 1), the equilibrium level of public good is normalised to one (ie e*_(*p = *1)_ = *c/u* = 1), entails *c* and *u* are expressed in units ensuring *c* = *u*.

Next we outline the stability conditions for the three fixed points of model (1). When *c* = *u*, the conclusions of the stability analysis described above for the one-dimensional model holds for the two-dimensional model. Generalising for any positive combination of *c* and *u*, the stability conditions for the three fixed points are as follows. Pure defection (p* = e* = 0) is locally stable if *S*<0. Pure cooperation (p* = 1, e* = *c/u*) is locally stable if *T*<1+*S*(*u-c*)/*c*. Finally, mixed cooperators and defectors (p* = *Su*/*c*(*S+T*-1), e* = *S*/(*S+T*-1)) is stable if *S*>0, *T*>1+*S*(*u-c*)/*c* and *S/*(*S+T*-1)<*u/c*. The approach to this equilibrium (when stable) will follow damped oscillations when the decay of the public good is sufficiently small, namely if *u*<*4cS*(*S+T*-1)/[*c*(*S+T*-1)*+*4*S* ^2^]. Under the constraint *c* = *u* = *x*, the condition for oscillations reduces to *x*<4*S*(*T*-1)/(*S*+*T*-1).

For any public good *e*, governed by parameters *S, T, u* and *c*, we have defined the scale of *e* so that when cooperators dominate (*p* = 1), the equilibrium level of public good equals one (ie e*_(*p = *1)_ = *c/u* = 1). But what if the relative strength of the parameters governing the production and decay of the public good (*c* and *u*) were to subsequently change? We can now ask what is the affect of a perturbation in the durability parameter *u*, relative to a reference game (pre-perturbation), defined by e*_(*p = *1)_ = *c/u* = 1. In the Snowdrift Game, decreasing the removal rate *u* (for constant *c*) has the expected consequence of reducing the equilibrium share of producers, as defectors are able to more effectively parasitize the durable public good (Fig. S1). Furthermore, we see that reducing *u* also has the effect of introducing oscillations (if *u*<*4cS*(*S+T*-1)/[*c*(*S+T*-1)+4*S* ^2^]) that become increasingly severe as the public good becomes long lasting. In the Stag-hunt Game, if we vary *u* independently of *c*, we see that decreasing *u* linearly reduces the repelling equilibrium point (Fig S2), potentially enlarging the basin of attraction for the pure cooperation equilibrium (cooperation has the highest payoff in the context of high levels of the public good, *e*). However, the extent to which the threshold to cooperator invasion is lowered depends on the history of cooperation, represented by the current value of the public good *e*. When *e* is absent (bottom of Fig S2), the threshold to cooperator invasion is raised, and altering the decay rate of the public good has only a minimal impact on the threshold position.

## Supporting Information

Figure S1 (0.04 MB PDF)

Figure S2 (0.05 MB PDF)

## References

[1] Hamilton WD (1964). The genetical evolution of social behaviour.. I. J Theor Biol.

[2] Trivers RL (1971). The evolution of reciprocal altruism.. Q Rev Biol.

[3] Wilson DS (1975). A theory of group selection.. Proc Natl Acad Sci U S A.

[4] Axelrod R (1984). The evolution of cooperation..

[5] Frank SA (1998). The foundations of Social Evolution..

[6] Sachs JL, Mueller UG, Wilcox TP, Bull JJ (2004). The evolution of cooperation.. Q Rev Biol.

[7] Hauert C, Doebeli M (2005). Models of cooperation based on the Prisoner's Dilemma and the Snowdrift Game.. Ecol Lett.

[8] Lehmann L, Keller L (2006). The evolution of cooperation and altruism-a general framework and a classification of models.. J Evol Biol.

[9] Nowak MA (2006). Five rules for the evolution of cooperation.. Science.

[10] Hauert C, Michor F, Nowak MA, Doebeli M (2006). Synergy and discounting of cooperation in social dilemmas.. J Theor Biol.

[11] Hauert C, Holmes M, Doebeli M (2006). Evolutionary games and population dynamics: maintenance of cooperation in public goods games.. Proc R Soc B.

[12] Foster KR, Wenseleers T (2006). A general model for the evolution of mutualisms.. J Evol Biol.

[13] Jansen VAA, van Baalen M (2006). Altruism through beard chromodynamics.. Nature.

[14] West SA, Griffin AS, Gardner A (2007). Social semantics: altruism, cooperation, mutualism, strong reciprocity and group selection.. J Evol Biol.

[15] Rapoport A, Geyer M, Gordon D (1976). The 2×2 Game..

[16] Maynard-Smith J, Price GR (1973). The logic of animal conflict.. Nature.

[17] Olson M (1965). The logic of collective action: public goods and the theory of groups..

[18] Hardin G (1968). The tragedy of the commons.. Science.

[19] Ostrom E (1990). Governing the Commons: The Evolution of Institutions for Collective Action..

[20] Dionisio F, Gordo I (2006). The tragedy of the commons, the public goods dilemma, and the meaning of rivalry and excludability in evolutionary biology.. Evol Ecol Res.

[21] Crespi BJ (2001). The evolution of social behavior in microorganisms.. Trends Ecol Evol.

[22] Brown SP, Johnstone RA (2001). Cooperation in the dark: signaling and collective action in quorum-sensing bacteria.. Proc R Soc B.

[23] Brown SP, Hochberg ME, Grenfell BT (2002). Does multiple infection select for raised virulence?. Trends Microbiol.

[24] Rainey PB, Rainey K (2003). Evolution of cooperation and conflict in experimental bacterial populations.. Nature.

[25] Grieg D, Travisano M (2004). The Prisoner's Dilemma and polymorphism in yeast SUC genes.. Proc R Soc B.

[26] Dugatkin LA, Perlin M, Lucas JS, Atlas R (2005). Group-beneficial traits, frequency-dependent selection and genotypic diversity: an antibiotic resistance paradigm.. Proc R Soc B.

[27] West SA, Griffin AS, Gardner A, Diggle SP (2006). Social evolution theory for microorganisms. Nature Rev Microbiol.

[28] Fehr E, Gächter S (2002). Altruistic punishment in humans.. Nature.

[29] Hofbauer J, Sigmund K (1998). Evolutionary games and population dynamics..

[30] Odling-Smee FJ, Laland KN, Feldma MW (2003). Niche Construction: the neglected process in evolution..

[31] Jones CG, Lawton JH, Shachak M (1994). Organisms as ecosystem engineers.. Oikos.

[32] Gardner A, West SA (2004). Cooperation and punishment, especially in humans.. American Naturalist.

[33] Nowak MA (2006). Evolutionary Dynamics: Exploring the Equations of Life..

[34] Brown SP, Le Chat L, De Paepe M, Taddei F (2006). Ecology of microbial invasions: amplification allows virus carriers to invade more rapidly when rare.. Current Biology.

