# Supplementary material for: The Durability of Public Goods Changes the Dynamics and Nature of Social Dilemmas
Source: PLoS One. 2007 Jul 4;2(7):e593. doi: 10.1371/journal.pone.0000593 (PMC1899228; doi:10.1371/journal.pone.0000593)

Figure S1. Snowdrift game (T =1.5, S = 0.5), public good (e) – cooperator (p) phase plane, independent rates of production and decay (c = 1, variable u), stable coexistence of cooperators and defectors at at ($p^* = (u/c)e^*$, $e^* = S/(S+T-1)$). Lines illustrate simulated trajectories for differing values of u (from 0.05 to 1), from initial position $p_0 = 0.3$, $e_0 = 0$.

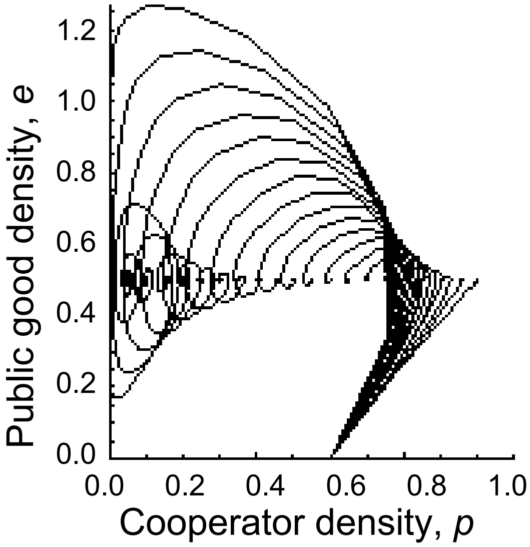

Supplement: Figure S1 — (0.04 MB PDF) [file pone.0000593.s001.pdf]
