# Supplementary material for: The Durability of Public Goods Changes the Dynamics and Nature of Social Dilemmas
Source: PLoS One. 2007 Jul 4;2(7):e593. doi: 10.1371/journal.pone.0000593 (PMC1899228; doi:10.1371/journal.pone.0000593)

Figure S2. Stag-hunt game (T =0.5, S = -0.5), public good (e) – cooperator (p) phase plane, independent rates of production and decay (c = 0.1, variable u). Lines illustrate simulated separatrices demarcating the basins of attraction for pure cooperator and pure defector equilibria (closed circles). Unstable equilibria (open circles) at ($p^* = (u/c)e^*$, $e^* = S/(S+T-1)$). Lines represent differing values of u (0.01, 0.1, 0.15). Note the line u = 0.1 corresponds to the line x = 0.1 in Fig. 3b.

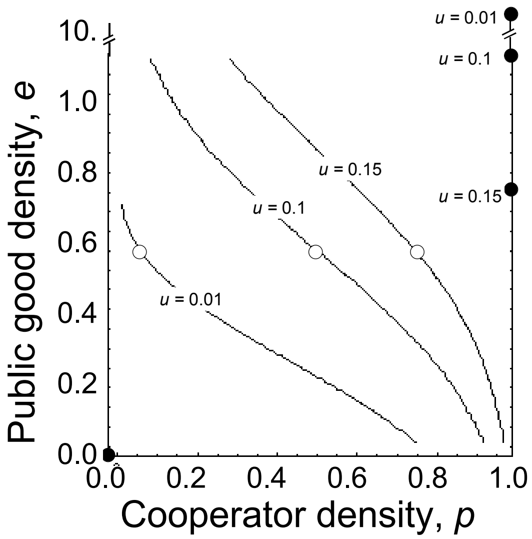

Supplement: Figure S2 — (0.05 MB PDF) [file pone.0000593.s002.pdf]
